# The Impact of Omitting Contralateral Systematic Biopsy on the Surgical Planning of Patients with a Unilateral Suspicious Lesion on Magnetic Resonance Imaging Undergoing Robot-assisted Radical Prostatectomy for Prostate Cancer

**DOI:** 10.1016/j.euros.2024.03.006

**Published:** 2024-03-21

**Authors:** Daniël L. van den Kroonenberg, Joëlle D. Stoter, Auke Jager, Hans Veerman, Marinus J. Hagens, Ivo G. Schoots, Arnoud W. Postema, Robert J. Hoekstra, Daniela E. Oprea-Lager, Jakko A. Nieuwenhuijzen, Pim J. van Leeuwen, André N. Vis

**Affiliations:** a Amsterdam UMC, location VUmc, Amsterdam, The Netherlands; b Prostate Cancer Network Netherlands, Amsterdam, The Netherlands; c Department of Urology, Netherlands Cancer Institute - Antoni van Leeuwenhoek Hospital, Amsterdam, The Netherlands; d Department of Radiology and Nuclear medicine, Erasmus MC, Rotterdam, The Netherlands; e Department of Radiology, Netherlands Cancer Institute - Antoni van Leeuwenhoek Hospital, Amsterdam, The Netherlands; f Department of Urology, Catharina Hospital, Eindhoven, The Netherlands; g Prosper Prostate Clinic, Nijmegen, The Netherlands; h Department of Radiology and Nuclear medicine, Amsterdam UMC, Amsterdam, The Netherlands

**Keywords:** Prostate cancer, Unilateral lesion, Magnetic resonance imaging, Perilesional biopsy, Prostate biopsy, Systematic biopsy, Targeted biopsy, Prostatectomy, Surgical planning

## Abstract

**Background and objective:**

A combined approach of magnetic resonance imaging (MRI)-targeted biopsy (TBx) and bilateral systematic biopsy (SBx) is advised in patients who have an increased risk of prostate cancer (PCa). The diagnostic gain of SBx in detecting PCa for treatment planning of patients undergoing robot-assisted radical prostatectomy (RARP) is unknown. This study aims to determine the impact of omitting contralateral SBx on the surgical planning of patients undergoing RARP in terms of nerve-sparing surgery (NSS) and extended pelvic lymph node dissection (ePLND).

**Methods:**

Case files from 80 men with biopsy-proven PCa were studied. All men had a unilateral suspicious lesion on MRI, and underwent TBx and bilateral SBx. Case files were presented to five urologists for the surgical planning of RARP. Each case file was presented randomly using two different sets of information: (1) results of TBx + bilateral SBx, and (2) results of TBx + ipsilateral SBx. The urologists assessed whether they would perform NSS and/or ePLND.

**Key findings and limitations:**

A change in the surgical plan concerning NSS on the contralateral side was observed in 9.0% (95% confidence interval [CI] 6.4-12.2) of cases. Additionally, the indication for ePLND changed in 5.3% (95% CI 3.3-7.9) of cases. Interobserver agreement based on Fleiss’ kappa changed from 0.44 to 0.15 for the indication of NSS and from 0.84 to 0.83 for the indication of ePLND.

**Conclusions and clinical implications:**

In our series, the diagnostic information obtained from contralateral SBx has limited impact on the surgical planning of patients with a unilateral suspicious lesion on MRI scheduled to undergo RARP.

**Patient summary:**

In patients with one-sided prostate cancer on magnetic resonance imaging, omitting biopsies on the other side rarely changed the surgical plan with respect to nerve-sparing surgery and the indication to perform extended lymph node dissection.

## Take Home Message

The diagnostic information obtained from contralateral systematic biopsies has a limited impact on the surgical planning of patients with a unilateral suspicious lesion on magnetic resonance imaging scheduled to undergo robot-assisted radical prostatectomy.

## 1 Introduction

The main surgical treatment option, in the western world, for men with localized prostate cancer (PCa) is robot-assisted radical prostatectomy (RARP). Owing to potential iatrogenic damage to the parasympathetic neurovascular bundles around the prostate, RARP is associated with a high risk of erectile dysfunction [1]. Preservation of the neurovascular bundle through nerve-sparing surgery (NSS) is the key to improving functional outcomes [1], [2]. An important factor to determine patient eligibility for NSS is the presence of extraprostatic extension (EPE) before surgery. Patients in whom EPE is expected are generally advised to refrain from NSS. The presence of EPE can be predicted using nomograms that incorporate tumor- and patient-specific parameters, such as prostate-specific antigen (PSA), clinical tumor stage (cT stage), and biopsy results [3], [4]. More recently, magnetic resonance imaging (MRI) has been integrated in EPE nomograms, showing a significant improvement in its predictive value [5]. Moreover, microultrasound has demonstrated even greater diagnostic performance than MRI in identifying EPE [6].

The introduction of MRI has impacted the diagnostic pathway for PCa. Performing MRI before prostate biopsy increased the detection of International Society of Urological Pathology (ISUP) grade group ≥2 PCa, while reducing the detection of ISUP 1 cancers [7]. Currently, the European Association of Urology (EAU) PCa guidelines recommend MRI targeted biopsy (TBx) combined with systematic biopsy (SBx) in all patients scheduled to undergo prostate biopsy for elevated serum PSA levels [8].

However, discussion remains whether the diagnostic information gained by bilateral SBx is clinically relevant in patients undergoing prostate biopsy. Combined TBx and SBx protocols appear to have largely similar detection rates for clinically significant PCa (csPCa) as biopsy protocols in which only TBx is performed. Performing only TBx without SBx lowers the diagnosis of ISUP 1 PCa while maintaining the detection of ISUP ≥2 PCa [9], [10], [11]. However, there are concerns that the lack of the diagnostic information gained from SBx could negatively impact the surgical planning of patients as well as surgical and oncological outcomes [12].

The objective of this study was to investigate whether the diagnostic information gained from SBx is of additional value in the surgical planning of patients scheduled to undergo RARP.

## 2 Patients and methods

A waiver of ethical approval and informed consent was provided previously by the Medical Research Ethics Committee Academic Medical Centre Amsterdam (MREC AMC, ID: W21_534 #21.590).

### 2.1 Study population

A total of 80 case files of consecutive patients who were formerly diagnosed with biopsy-proven PCa were evaluated (Table 1). Patients were included if they had a unilateral suspicious lesion (Prostate Imaging Reporting and Data System [PI-RADS] ≥3) on MRI and if they underwent both TBx and bilateral SBx. PCa was diagnosed at Amsterdam University Medical Centers between August 2018 and January 2023.

**Table 1:** Clinicopathological and radiological characteristics of the patients who were diagnosed with prostate cancer

Prebiopsy serum PSA (ng/ml), median (IQR)	6.9 (5.2-10)	
PSA density, median (IQR)	0.17 (0.12-0.30)	
Digital rectal exam a,*N* (%)		
Benign	34 (43)	
Malignant	38 (48)	
Dubious	6 (7.5)	
Missing	2 (2.5)	
MRI result, *N* (%)		
PI-RADS 3	7 (8.8)	
PI-RADS 4	35 (44)	
PI-RADS 5	37 (46)	
Radiological tumor stage, *N* (%)		
mT2	58 (73)	
mT3a	18 (23)	
mT3b	4 (5.0)	
Pathology results	Bilateral SBx + TBx	Ipsilateral SBx + TBx
Highest ISUP, *N* (%)		
ISUP 1	0	0
ISUP 2	35 (48)	37 (46)
ISUP 3	23 (29)	21 (26)
ISUP 4	6 (7.5)	6 (7.5)
ISUP 5	16 (20)	16 (20)
Positive biopsy cores, mean (% of total biopsies)	7 (47)	6 (67)
Presence of cribriform growth a,*N* (%)		
Yes	29 (36)	
No	18 (23)	
Missing	33 (41)	

IQR = interquartile range; ISUP = International Society of Urological Pathology; MRI = magnetic resonance imaging; PI-RADS = Prostate Imaging Reporting and Data System; PSA = prostate-specific antigen; SBx = systematic biopsy; TBx = target biopsy.

a Information known only for bilateral SBx +TBx results.

### 2.2 MRI protocol

MRI image acquisition was performed according to the PI-RADS V2.1 guidelines, using either a 1.5 Tesla AVANTO MRI scanner (Siemens Healthcare, Erlangen, Germany) or a 3 Tesla INGENIA MRI scanner (Philips Medical Systems, Best, the Netherlands) [13]. MRI sequences included at least T1-weighted, T2-weighted, and diffusion-weighted imaging and apparent diffusion coefficient maps.

### 2.3 Biopsy protocol and histopathological assessment

Experienced operators performed transrectal prostate biopsy until July 2020 and then switched to the transperineal approach. Transrectal prostate biopsy was conducted using the Philips iU-22 ultrasound (US) system with an end-firing probe. Biopsy generally included 12-core SBx and two- to three-core TBx per suspicious MRI lesion [10]. Transrectal TBx was facilitated by the MRI/US-fusion software of ProFuse in combination with the Artemis fusion system.

The BK5000 ultrasound system (BK Medical Europe, Herley, Denmark) with a biplane probe was used to perform transperineal prostate biopsy. The probe was secured on a stabilizer and stepper, and the biopsy was performed using a brachytherapy template grid. Biopsy usually consisted of 14-core SBx and two- to three-core TBx per suspicious MRI lesion. MIM (MIM Software Inc., Cleveland, OH, USA) integrated elastic MRI/US-fusion software was used for transperineal TBx.

Biopsy cores were evaluated by different uropathologists according to the ISUP grade group consensus for the grading of PCa [14].

### 2.4 Assessment of the surgical plan using case files

Five robotic urologists from three centers, with 1-15 yr of experience in RARP, independently made a surgical plan for different phases of RARP based on case files presented as preoperative available data. These case files contained clinical data (PSA, PSA density, and cT stage), MRI results (prostate volume, location of lesion, PI-RADS classification, and radiological T stage), biopsy pathology (location, number of cores positive for PCa, and ISUP grade), and risk prediction for lymph node metastatic disease using the Briganti 2019 nomogram based on TBx and bilateral SBx results (Table 1 and Supplementary Fig. 1) [15]. The observers made a surgical plan with respect to NSS (none, ipsilateral, contralateral, or bilateral) and whether they would perform extended pelvic lymph node dissection (ePLND).

From the 80 included patients, 160 case files were created, with 80 having bilateral biopsy results and 80 without. Case files were presented randomly to the urologist over a period of 6 wk, in four rounds of 40 cases. The original scenario and the study scenario were presented at least 2 wk apart, to prevent bias. In the first scenario, the surgical planning was based on the diagnostic information obtained from both TBx and bilateral SBx. In the second scenario, the surgical plan was based solely on the diagnostic information from TBx and ipsilateral SBx. In this second scenario, the observer was blinded to the results of the contralateral SBx results. It was assumed that all patients had good erectile function prior to surgery and opted for maximal nerve preservation if possible.

### 2.5 Statistical analysis

Descriptive analyses were performed using STATA (version 16). Non-normally distributed data are presented as median and interquartile range (IQR). Statistical differences between medians were calculated using the Wilcoxon matched-pair signed-rank test. A *p* value of <0.05 was considered statistically significant. Interobserver agreement is expressed as Fleiss’ kappa, and intraobserver agreement is expressed as Cohen’s kappa. Kappa values are interpreted as follows: poor agreement (0.00-0.40), fair agreement (0.40-0.75), or excellent (0.75-1.00) [16].

## 3 Results

Patient characteristics, radiological outcome, and biopsy results are presented in Table 1.

A median of three biopsies (IQR 3-3) were taken for TBx, and a median of six biopsies (IQR 6-7) per side of the prostate were taken for SBx.

### 3.1 Highest ISUP grading according to diagnostic scenarios

When TBx and bilateral SBx were performed, 35 (45%), 23 (29%), six (7.5%), and 16 (20%) men had ISUP 2, 3, 4, and 5 PCa on biopsy, respectively. These figures were, respectively, 37 (46%), 21 (26%), six (7.5%), and 16 (20%) if contralateral biopsy results were not available (*p* = 0.50). In 27 (34%) patients, csPCa was found in the contralateral SBx. The ISUP scores of the contralateral SBx were 19 (24%) for ISUP 1, 17 (21%) for ISUP 2, 5 (6.2%) for ISUP 3, 3 (3.8%) for ISUP 4, 2 (2.5%) for ISUP 5, and benign in 34 (43%) cases. In only two (2.5%) cases, ISUP grade was higher in the contralateral SBx than in the TBx + ipsilateral SBx biopsy. In these cases, ISUP 3 was found, and TBx and ipsilateral SBx results showed ISUP 2.

### 3.2 Nerve-sparing surgery

Among patients for whom a surgical strategy was planned based on the diagnostic information from TBx and bilateral SBx, an NSS approach was chosen in 364 cases (91%; 95% confidence interval [CI] 88-94). Of these cases, 177 (44%) had one-sided NSS and 187 (47%) had two-sided NSS. Of patients in whom a surgical plan was made based on the diagnostic information of TBx and ipsilateral SBx, 370 (95%; 95% CI 90-95) were selected for NSS (*p* = 0.50). Of these cases, 174 (44%) had one-sided NSS and 196 (49%) had two-sided NSS. The total number of changes to the NSS plan per urologist depending on the biopsy strategy used (TBx + bilateral SBx vs TBx + ipsilateral SBx) varied from 1 (1.3%) to 24 (30%) out of 80 cases. Table 2 illustrates the changes in the NSS strategy for each urologist.

**Table 2:** Surgical planning of nerve-sparing surgery by different observers

	Bilateral + contralateral NSS		Change when leaving out contralateral biopsy results		Difference	Cohen’s kappa
	With result from contralateral SBx	Without result from contralateral SBx	Yes to no	No to yes		
Urologist 1, *n* (%)	76 (95)	77 (96)	0	1 (1.3)	1 (1.3)	0.85
Urologist 2, *n* (%)	74 (93)	79 (99)	0	5 (6.3)	5 (6.3)	0.27
Urologist 3, *n* (%)	76 (95)	75 (94)	2 (2.5)	1 (1.3)	3 (3.8)	0.65
Urologist 4, *n* (%)	65 (81)	63 (79)	13 (16)	11 (14)	24 (30)	0.06
Urologist 5, *n* (%)	73 (91)	76 (95)	0	3 (3.8)	3 (3.8)	0.71
Overall *n* (%, 95% CI)	364 (91%, 88-94)	370 (93%, 90-95)	15 (3.8%, 2.1-6.1)	21 (5.3%, 3.3-7.9)	36 (9.0%, 6.4-12)	
Fleiss’ kappa	0.44	0.15				

CI = confidence interval; NSS = nerve-sparing surgery; RARP = robot-assisted radical prostatectomy; SBx = systematic biopsy.

The assessments of different urologists in the surgical planning of patients undergoing RARP were based on a subset of clinical, radiological, and pathological clinical file data. The percentages point out in which patients contralateral or bilateral NSS would be performed with and without the diagnostic information obtained from contralateral SBx. Intraobserver agreement is expressed as Cohen’s kappa, and interobserver agreement is expressed as Fleiss’ kappa.

Interobserver agreement, as measured by Fleiss’ kappa, was fair to good (0.44) when bilateral SBx and TBx were presented, and also poor (0.15) when ipsilateral SBx and TBx were presented. Intraobserver agreement, as measured by Cohen’s kappa, was poor (0.06) to excellent (0.85; Table 2).

### 3.3 Extended pelvic lymph node dissection

When the diagnostic information was available for both TBx and bilateral SBx, ePLND was chosen in 227 cases (57%; 95% CI 51-62). This figure was 228 (57%; 95% CI 52-62) when the surgical plan was based on the diagnostic information obtained by TBx and ipsilateral SBx only (*p* = 0.89). The number of differences in the surgical plan to perform ePLND per urologist depending on the biopsy strategy used (TBx + bilateral SBx vs TBx + ipsilateral SBx) varied from 3 (3.8%) to 5 (6.3%) out of 80 cases. Table 3 illustrates the difference in ePLND for each urologist, as well as the change in the ePLND strategy.

**Table 3:** Surgical planning of ePLND by different observers

	ePLND plan		Change when leaving out contralateral biopsy results		Difference	Cohen’s kappa
	With result from contralateral SBx	Without result from contralateral SBx	Yes to no	No to yes		
Urologist 1, *n* (%)	44 (55)	49 (61)	0	5 (6.3)	5 (6.3)	0.87
Urologist 2, *n* (%)	48 (60)	47 (59)	2 (2.5)	1 (1.3)	3 (3.8)	0.92
Urologist 3, *n* (%)	45 (56)	42 (53)	4 (5.0)	1 (1.3)	5 (6.3)	0.87
Urologist 4, *n* (%)	48 (60)	49 (61)	2 (2.5)	3 (3.8)	5 (6.3)	0.87
Urologist 5, *n* (%)	42 (52)	41 (51)	2 (2.5)	1 (1.3)	3 (3.8)	0.92
Overall *n* (%, 95% CI)	227 (57%, 51-62)	228 (57%, 52-62)	10 (2.5%, 1.2-4.5)	11 (2.8%, 1.4-4.9)	21 (5.3%, 3.3-7.9)	
Fleiss’ kappa	0.84	0.83				

CI = confidence interval; ePLND = extended pelvic lymph node dissection; RARP = robot-assisted radical prostatectomy; SBx = systematic biopsy.

The assessments of different urologists in the surgical planning of patients undergoing RARP were based on a subset of clinical, radiological and pathological file data. The percentages point out in which patients ePLND would be performed with and without the diagnostic information obtained from contralateral SBx. The change points out in which direction the surgical plan for ePLND differs if diagnostic information from contralateral SBx is unavailable. Intraobserver agreement is expressed as Cohen’s kappa, and interobserver agreement is expressed as Fleiss’ kappa.

The median risks of lymph node involvement based on the Briganti 2019 nomogram were 17% (95% CI 3-29) and 21% (95% CI 4-42) for patients with TBx and ipsilateral SBx results, respectively. In 10% of cases, this risk increased above the validated cutoff of 7% if only ipsilateral SBx and TBx results were known.

Interobserver agreement based on Fleiss’ kappa was excellent in both scenarios regarding the ePLND strategy. The kappa was 0.84 for the scenario where bilateral SBx and TBx results were available, and 0.83 for the scenario where only ipsilateral SBx and TBx results were available (Table 3). Intraobserver agreement based on Cohen’s kappa was excellent (0.87-0.92) for all urologists.

## 4 Discussion

At present, the EAU guidelines advise TBx and bilateral SBx in all patients at an increased risk of PCa for whom prostate biopsy is indicated [8]. Although this combined biopsy approach is the recommended method, the efficacy and relevance of SBx have become a subject of debate [17]. The diagnostic benefits of this approach may be questionable, but it could contribute to treatment planning.

This study evaluated the relevance of contralateral SBx for the surgical planning in 80 consecutive patients with a unilateral suspicious lesion on diagnostic MRI undergoing RARP for PCa. We found that omitting contralateral SBx in men with a unilateral suspicious lesion on MRI had only a minimal impact on the rate of NSS planned. The rate of NSS changed according to the available diagnostic information of SBx in 36 (9.0%) out of 400 cases. Our study further showed that in terms of the indication for ePLND, the surgical plan changed in 21 (5.3%) out of 400 cases when additional diagnostic information of contralateral SBx was available. Therefore, the diagnostic dilemma is thrown up whether contralateral SBx is truly relevant for clinical decision-making, and thus, whether it could be omitted safely in those in whom prostate biopsy is prompted.

The omission of contralateral SBx in patients with a unilateral suspicious lesion on MRI is not without risks. It is assumed that if the diagnostic information of contralateral SBx is not present, urologists may choose for an NSS approach slightly more often than in the scenario in which the diagnostic information of contralateral SBx is available. This is particularly true if csPCa is present in one or more of these contralateral SBx cases despite being nonsuspect on diagnostic MRI. NSS is associated with higher positive surgical margin rates and may be particularly concerning if intermediate- or high-grade disease is present at the margin of resection [18]. We showed in our series that in 34% of cases, csPCa was present on the contralateral side of the prostate. However, according to Soeterik et al’s [4] nomogram, the median probability of side-specific EPE on the contralateral side was 18% (IQR 14-25). Applying the validated cutoff of 20% for the indication of NSS could suggest the performing contralateral NSS without contralateral SBx results carries limited risks of EPE in these series.

More importantly, availability of the diagnostic information from contralateral SBx changed the surgical plan only rarely, even when csPCa was present in these biopsies. Regarding the decision to perform NSS, we found variation between urologists, for both diagnostic scenarios (Table 2). One urologist made notably more changes to the NSS plan, which could be explained by the fact that he had only 1 yr of experience in performing RARP surgery.

Similar results were found regarding the indication for ePLND. Being informed about the biopsy outcome of contralateral SBx biopsy rarely changed the indication for ePLND. Only if intermediate- or high-grade disease was detected in the contralateral prostate biopsies, the decision to refrain from ePLND could have been refuted and changed into a surgical plan in which ePLND was performed. The five urologists had high interobserver agreement.

In this study, ipsilateral biopsies were performed as part of SBx. These biopsies differ from the perilesional biopsies studied by Hagens et al [10]. Perilesional biopsies are taken from the prostate tissue surrounding an MRI lesion, whereas ipsilateral SBx is taken from one half of the prostate, mostly the peripheral zone. In practice, the number and location of these strategies are comparable. Perilesional biopsies have been shown to be an interesting strategy with equivalent csPCa detection to SBx and TBx, while reducing the total number of biopsies taken [7], [10], [11].

Although the sensitivity of MRI for csPCa at a patient level is generally high, it is considerably lower at a per-lesion level, particularly for smaller and less aggressive tumors [7], [19], [20]. Bilateral SBx can function as a safety net for these missed csPCa lesions. In other words, additional csPCa is indeed detected by performing more biopsies than TBx only [21]. On the contrary, bilateral SBx leads to increased levels of pain, discomfort, and hematuria compared with TBx and contributes to overdiagnosis [22].

With respect to the surgical planning of patients who opt for RARP, the presence of contralateral csPCa does not always change the surgical plan. In our series, approximately one-third of patients had csPCa on the contralateral side, mostly attributed to the presence of low-volume ISUP 2 lesions. Apparently, the presence of low-volume ISUP 1 and 2 lesions did not make the observers decide to change from NSS to non-NSS, or to change the surgical plan regarding ePLND.

There are several limitations to this study. First, the study has a retrospective design and is based on hypothetical treatment planning rather than actual treatment. Ideally, the actual impact of omitting contralateral SBx on oncological and functional outcomes after RARP would be better investigated in a prospective or randomized controlled design, which is less likely to be performed. Second, MRI results were not revised centrally. Given the variability in the diagnostic performance of MRI between observers and between different centers performing MRI, this may have influenced the results of the current study [13], [23]. However, all the treatment and surgical plans for both diagnostic scenarios were based on the same case files and the same MRI scans.

## 5 Conclusions

The diagnostic information obtained from contralateral SBx has limited impact on the surgical planning of patients with a unilateral suspicious lesion on MRI scheduled to undergo RARP. In few patients, the surgical plan changed when the diagnostic information of contralateral SBx was known. In our series, only 3% of patients were found to have a higher-grade tumor in contralateral SBx than in ipsilateral SBx or TBx.

  ***Author contributions*:** Daniël L. van den Kroonenberg had full access to all the data in the study and takes responsibility for the integrity of the data and the accuracy of the data analysis.

  *Study concept and design*: van den Kroonenberg, Stoter, Jager, Vis.

*Acquisition of data*: van den Kroonenberg, Stoter.

*Analysis and interpretation of data*: van den Kroonenberg, Stoter, Jager, Vis.

*Drafting of the manuscript*: van den Kroonenberg, Stoter, Jager, Vis.

*Critical revision of the manuscript for important intellectual content*: van den Kroonenberg, Stoter, Jager, Veerman, Hagens, Schoots, Postema, Hoekstra, Oprea-Lager, Nieuwenhuijzen, van Leeuwen, Vis.

*Statistical analysis*: van den Kroonenberg, Stoter, Jager.

*Obtaining funding*: None.

*Administrative, technical, or material support*: None.

*Supervision*: Vis.

*Other*: None.

  ***Financial disclosures:*** Daniël L. van den Kroonenberg certifies that all conflicts of interest, including specific financial interests and relationships and affiliations relevant to the subject matter or materials discussed in the manuscript (eg, employment/affiliation, grants or funding, consultancies, honoraria, stock ownership or options, expert testimony, royalties, or patents filed, received, or pending), are the following: None.

  ***Funding/Support and role of the sponsor*:** None.
