# Supplementary material for: The Impact of Omitting Contralateral Systematic Biopsy on the Surgical Planning of Patients with a Unilateral Suspicious Lesion on Magnetic Resonance Imaging Undergoing Robot-assisted Radical Prostatectomy for Prostate Cancer
Source: Eur Urol Open Sci. 2024 Mar 21;63:13–8. doi: 10.1016/j.euros.2024.03.006 (PMC10981034; doi:10.1016/j.euros.2024.03.006)
Supplement: Supplementary data 1 [file mmc1.docx]

**Supplementary Figure 1**

Supplementary Figure 1A. Example of a patient case in which clinical chart data (PSA, PSA-density, clinical tumor stage, prostate volume), radiological data (PI-RADS score, location of tumor, radiological tumor stage, T2 and ADC weighted images) and the calculated Briganti 2019 nomogram for the risk of lymph-node metastatic disease were presented to a urologist performing RARP (1). The representative images of the MRI were presented as well. Using these clinical file data, the urologist made a surgical plan on nerve-sparing surgery and the indication for ePLND.


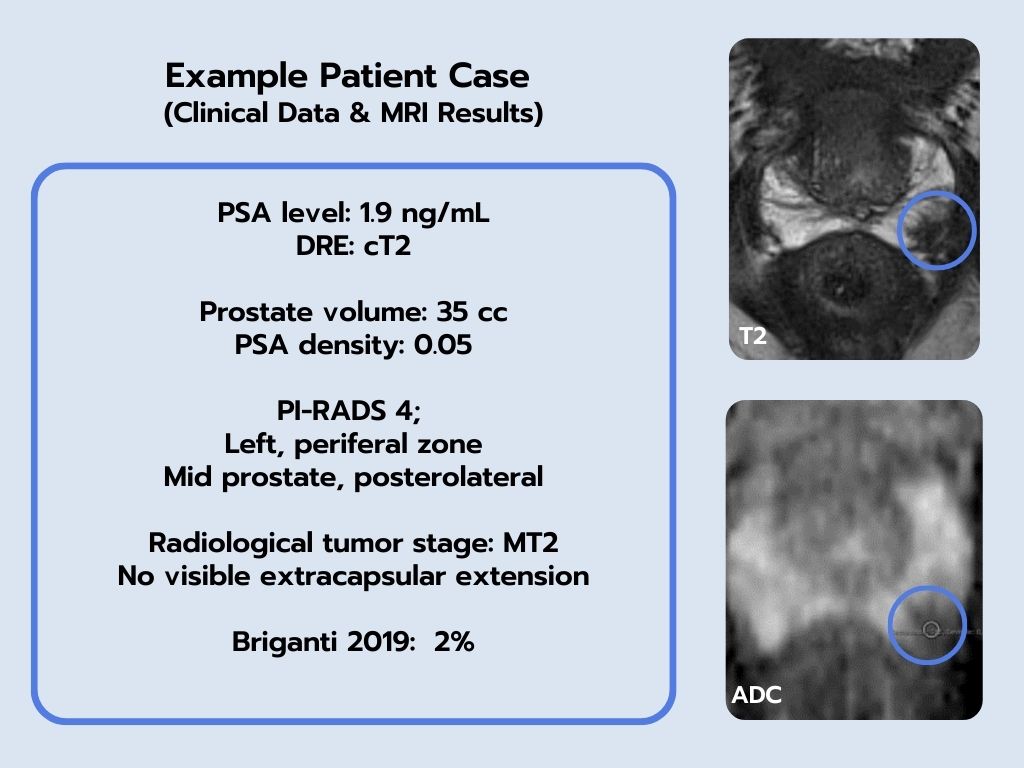


PSA: prostate specific antigen; DRE: digital rectal examination; RARP: robot-assisted radical prostatectomy;
ePLND: extended pelvic lymph node dissection, ADC; apparent diffusion coefficient

1. Gandaglia G, Ploussard G, Valerio M, Mattei A, Fiori C, Fossati N, et al. A Novel Nomogram to Identify Candidates for Extended Pelvic Lymph Node Dissection Among Patients with Clinically Localized Prostate Cancer Diagnosed with Magnetic Resonance Imaging-targeted and Systematic Biopsies. Eur Urol. 2019;75(3):506-14.

Supplementary Figure 1B. Diagnostic information from TBx and bilateral SBx . Secondly, the urologist was asked to make a surgical plan based on the data obtained from Figure 1a as well as on the outcome of prostate biopsies (TBx and bilateral SBx) including the number of positive for cancer biopsies and the ISUP grades.


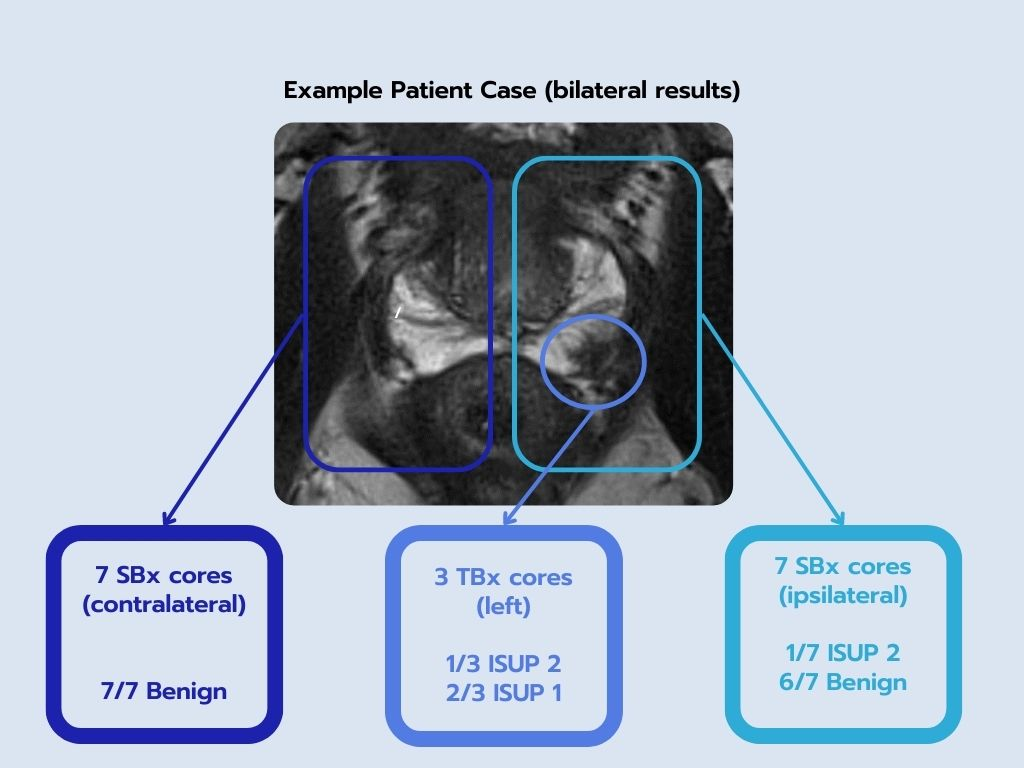


TBx: target biopsies; SBx systematic biopsies; ISUP: International Society of Urological Pathology

Supplementary Figure 1C. Diagnostic information from TBx and ipsilateral SBx only. Thirdly, the urologist was asked to make a surgical plan based on the data obtained from Figure 1a as well as on the outcome of prostate biopsies (TBx and ipsilateral SBx only) including the number of positive


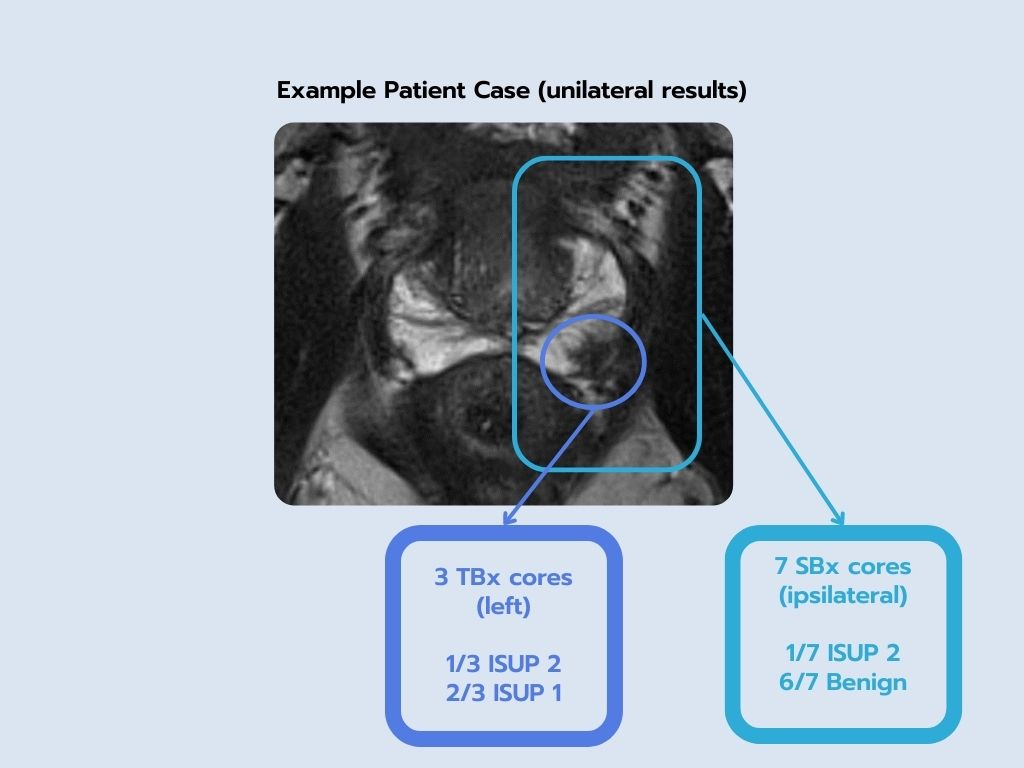
for cancer biopsies and the ISUP grades.

TBx: target biopsies; SBx: systematic biopsies; ISUP: International Society of Urological Pathology
